# Perceived social support, fatigue, and sleep quality in women treated for gynecological cancer: a cross-sectional study

**DOI:** 10.1007/s00520-025-09674-5

**Published:** 2025-06-28

**Authors:** Nurcan Kirca, Derya Adibelli, Tayfun Toptas, Senay Yilmaz

**Affiliations:** 1 Obstetrics and Gynecology Nursing Department, Faculty of Nursing, Akdeniz University, Antalya, Turkey; 2 Public Health Nursing Department, Antalya Science University Faculty of Health Sciences, Antalya, Turkey; 3 University of Health Sciences Antalya Research and Training Hospital, Antalya, Turkey

**Keywords:** Fatigue, Gynecological oncology, Sleep quality, Social support, Nursing

## Abstract

**Aim:**

The study aims to determine the levels of perceived social support, fatigue, and sleep quality among women undergoing treatment for gynecological cancer.

**Methods:**

This cross-sectional and descriptive study included 217 women. Participants completed the personal information form, the multidimensional perceived social support scale, the Pittsburgh Sleep Quality Index, and the brief fatigue questionnaire. This study was conducted with patients who received treatment at the Gynaecological Oncology Clinic of a Training and Research Hospital and met the inclusion criteria. Data were collected between April and September 2023 after obtaining approval from the ethics committee and the relevant institution.

**Results:**

The mean total score for perceived social support was 59.85 ± 17.04, the median total score on the fatigue scale was 63.00 (IQR = 16.50), and the mean total score on the PSQI was 6.91 ± 2.60. Most of the participants (67.4%) had poor sleep quality. There was a weak negative correlation between the daytime dysfunction subdimension and the total PSQI score and both the subdimensions and total score of the social support scale, as well as a weak positive correlation with the fatigue scale (*p* < 0.05).

**Conclusion:**

In our study, perceived social support and fatigue scores among patients with gynecological cancer were found to be above the moderate level, while their sleep quality was poor.

## Introduction

Gynecological cancers are of critical importance as they significantly impact women’s lives and health. In 2020, more than 1.3 million new cases of gynecological cancer and 671,875 related deaths were reported worldwide [1]. The incidence and mortality rates of these cancers continue to rise globally [1, 2]. Common types include cervical, ovarian, endometrial, and vaginal cancers [3–6].

The quality of life of individuals is adversely affected by the physical, psychological, and socioeconomic challenges associated with cancer and its treatment, leading to reduced sleep quality and increased levels of fatigue. Social support has a positive influence on health outcomes [7] and quality of life in patients with gynecological cancer. It can help patients to adapt to the disease and treatment process and prolong their life span [8]. The presence or absence of social support may be an important factor influencing the development and progression of cancer. Social support helps cancer patients feel that others care about them and accept them. The existence of supportive interpersonal relationships has the potential to positively influence cancer-related well-being [9]. The multifaceted nature of social support, encompassing emotional assistance and information sharing, emerges as a pivotal factor aiding patients in confronting the challenges inherent to gynecological cancer [10]. This form of support contributes to reducing fatigue and enhancing overall sleep.

The disease and treatment process may cause fatigue in patients diagnosed with cancer [11]. Fatigue is perceived by individuals with cancer as a subjective symptom characterized by weakness, impaired concentration, lack of strength and motivation, insomnia, decreased sleep quality, distress, low energy levels, and an increased need for rest [12]. It has been reported that individuals diagnosed with cancer who experience fatigue tend to reduce vital activities, such as altering their daily routines and spending less time walking [11]. Long-term fatigue is reported to be one of the most common and distressing side effects of cancer and its treatment [13]. Most individuals diagnosed with cancer have a diagnosis of fatigue, and it persists for a long time afterwards [13]. In a study on fatigue, cancer patients receiving chemotherapy and radiotherapy were examined, and it was concluded that fatigue affects spiritual well-being, and spiritual well-being was positively affected by decreased fatigue [11]. Studies have shown that fatigue is a significant and commonly experienced problem among cancer patients [14, 15].

Sleep disturbance is a common yet frequently overlooked problem among cancer patients. Sleep disturbance is often overlooked in cancer patients because its effects are considered normal or temporary during diagnosis and treatment, and individuals may not report these issues to healthcare professionals. Sleep disturbance may be caused by cancer, type and stage of cancer, and side effects. Insomnia can often be severe enough to require medical treatment [16]. Although sleep disturbance is known to increase mortality in cancer patients, treating insomnia is essential for maintaining psychophysical health.

A positive relationship has been reported between sleep disturbance and fatigue in individuals with cancer [17]. In cancer patients, sleep disturbance is more prevalent among those who report fatigue complaints [17]. In one study, sleep quality was assessed before and after chemotherapy, and a decrease in sleep duration was observed. Based on the findings, it was recommended that sleep quality be evaluated throughout the treatment period [18].

Sleep disorders are one of the signs of fatigue. Fatigue, poor sleep quality, difficulty falling and staying asleep, insufficient sleep, and non-restorative sleep are interrelated in cancer patients [19]. Fatigue and decreased sleep quality are also prevalent among patients with gynecological cancer, which is common both globally and in Turkey [19]. Although changes in fatigue and sleep quality caused by cancer and affecting patients’ quality of life can be recognised and assessed by nurses and other healthcare professionals [19, 20], fatigue and sleep disturbances resulting from complications are often overlooked by both patients and healthcare providers. Social support, fatigue, and sleep quality should be carefully considered and addressed to enhance the treatment process, manage the disease course, and improve the patient’s quality of life.

Since there are not enough studies on this subject, this study was conducted to evaluate gynecological cancer patients by nurses within the scope of holistic care, to consider perceived social support, fatigue, and sleep quality, to provide adequate nursing care and to examine whether there is a relationship between them. Due to the limited number of studies on this subject, this study was conducted to evaluate patients with gynecological cancer within the scope of holistic nursing care, to consider perceived social support, fatigue, and sleep quality, to provide appropriate nursing interventions, and to examine the relationships among these variables.

Study questions:

- What is the level of perceived social support among women diagnosed with gynecological cancer?
- What is the fatigue level of women diagnosed with gynecological cancer?
- What is the sleep quality level of women diagnosed with gynecological cancer?
- Is there a relationship between perceived social support, fatigue, and sleep quality among women diagnosed with gynecological cancer?

## Methods

### Study design and participants

A descriptive cross-sectional design was employed. The study population consisted of patients receiving treatment at the Gynaecological Oncology Clinic of a Training and Research Hospital between April and September 2023. Inclusion criteria: (1) age ≥ 18 years; (2) having a diagnosis of gynecological cancer; (3) only primary gynecological cancer patient; (4) with cancer in stages I, II, III, or IV; (5) being in the treatment process; (6) volunteering to participate in the study; (7) being able to understand and speak Turkish; (8) and not having any psychiatric disorder.

Exclusion criteria: (1) insufficient awareness of the disease, (2) severe concurrent systemic diseases, (3) secondary gynecological cancer patient, (4) other types of cancer.

### Sample size

The sample size was estimated by a power analysis based on *α* = 0.05 and power of the test of 1-*β* = 0.80; a sample size of 187 patients was required to achieve 90% power. Over a 6-month period, a convenience sample of 217 patients was recruited for the study. All participants met the study inclusion criteria and agreed to participate, resulting in a 100% response rate.

#### Data collection

Data were collected between April and September 2023 after obtaining approval from the ethics committee and the relevant institution. Patients who met the study inclusion criteria were invited to participate in the study. The researcher conducted face-to-face interviews with the patients who participated in the study. The aim of the study was explained to eligible participants, and informed consent was obtained prior to participation. Data were collected through face-to-face interviews with all participants. The participants completed the questionnaires themselves in approximately 15 min. All participant data were collected in accordance with the principles of confidentiality.

### Forms used for data collection

Data were collected using the personal information form, the multidimensional perceived social support scale, the Pittsburgh Sleep Quality Index, and the brief fatigue questionnaire.

### Personal information form

The form includes sociodemographic information such as age, educational status, employment status, perceived income level, type of cancer (ovarian, endometrial, cervical), time since diagnosis (in months), stage of the disease, and descriptive details regarding the duration of gynecological diagnosis and treatment.

### Multidimensional perceived social support scale (MPSSS)

The validity and reliability of the scale was conducted by Eker et al. [21]. The scale is a 7-point Likert-type scale and consists of 3 sub-dimensions (family, friend, and special person support) and 12 items. Family support: consists of items 3, 4, 8, and 11 of the scale. Friend support: consists of items 6, 7, 9, and 12 of the scale. Special person support: consists of items 1, 2, 5, and 10 of the scale. The lowest score that can be obtained from the total scale is 12 and the highest score is 84; the lowest score that can be obtained from the sub-dimensions of the scale is 4 and the highest score is 28. A high score obtained from the scale indicates that the perceived social support is high. Eker et al. [21] reported that the sub-dimensions and total Cronbach’s alpha coefficient of the scale were between 0.77 and 0.92. In this study, the Cronbach’s alpha coefficient of the family sub-dimension of the scale was 0.82, the friend’s sub-dimension was 0.94, the private person support sub-dimension was 0.96, and the total scale was 0.94.

### Pittsburgh Sleep Quality Index (PSQI)

The validity and reliability of the scale were performed by Agargun et al. [22]. The scale consists of a total of 19 questions and 7 components. These components are subjective sleep quality, sleep latency, sleep duration, habitual sleep efficiency, sleep disturbance, sleep medication use, and daytime dysfunction. Eighteen items and 7 components are included in the scoring. Each item is evaluated on a 0–3 point scale and the sum of the 7 component scores gives the total PSQI score. The total score is between 0 and 21. A high score indicates poor sleep quality and a total score of ≤ 5 indicates “good sleep quality” and > 5 indicates “poor sleep quality.” In this study, the Cronbach’s alpha coefficient was found to be 0.96.

### Brief fatigue inventory

The validity and reliability study of the scale was conducted by Azak and Çınar [23]. It consists of 10 questions. The scale begins with a section that assesses whether individuals with cancer have experienced fatigue differently than usual in the past week, with responses given as yes or no. Based on the data obtained from this section, the percentage of fatigue in the study group is determined. The form assesses general fatigue levels—such as fatigue experienced at the time of the interview, overall fatigue in the past 24 h, and the most severe fatigue level in the past 24 h—as well as the extent to which fatigue has affected daily activities in the past 24 h, including general activity, mood, walking ability, life outside the home, interactions with others, and enjoyment of life. The arithmetic mean of the scores from the first three questions is calculated to determine each individual’s overall fatigue score. Scoring is done between 0 and 10, with “0” indicating not being affected at all and “10” indicating being affected at the highest level. Accordingly, 0 points: no fatigue at all 1–2: fatigue at a very low level 3–4: fatigue at a low level 5–6: fatigue at a medium level 7–8: fatigue at a high level 9–10: fatigue at a very high level. The score assigned to the options in the fourth question—which covers general activity, mood, walking ability, work outside the home, communication with others, and joy of living—indicates the extent to which these aspects are affected by the fatigue experienced by the individual. In the study conducted by Azak and Çınar [23] on hematological cancer patients, it was found to be 0.98. In this study, it was found to be 0.87.

### Analysis

The study data were analyzed using arithmetic mean, standard deviation, median, interquartile range (IQR), skewness and kurtosis coefficients, one-way analysis of variance (ANOVA), Kruskal–Wallis test, LSD and Tamhane’s tests for post hoc analyses, and correlation analyses, all conducted using the Statistical Package for the Social Sciences (SPSS) version 25.0. A skewness value within the range of ± 1.5 indicates that the data conform to a normal distribution. It was determined that the sleep duration subdimension of the Pittsburgh Sleep Quality Index (PSQI) and the fatigue scale did not follow a normal distribution, whereas all other subdimensions and the subdimensions of the social support scale were normally distributed.

*Ethical consideration.*

Ethics committee approval was obtained from Akdeniz University Clinical Research Ethics Committee (Approval number: 218, Date: 08 March 2023). The study was carried out according to the principles of the Declaration of Helsinki. All participants were informed that they could withdraw from the study at any time, and written informed consent was obtained.

## Results

### Sample characteristics

The mean age of the women in the study was 52.94 ± 0.73, and the mean and median scores of the scales and their subdimensions are presented in Table 1. Of the participants, 79.7% were married, 37.8% were high school graduates, and 80.6% had a nuclear family structure. The majority (87.1%) lived with their families, and more than half (50.2%) were not employed. Most of them lived in the city center (46.5%) and stated that their income was less than their expenses (74.2%). More than half stated that they were informed about the disease (78.3%) and treatment (79.7%). Endometrium cancer (49.8%) was the most common diagnosis among the participants. A percentage of 32.7 stated that their disease was in the third stage. Surgical treatment was performed in more than half (51.6%). Most of the participants (67.4%) had poor sleep quality (Table 1).

**Table 1:** Demographic and clinical characteristics of the participants

Sample characteristics	*n*	(%)
Average age 52.94 ± 0.73 (min–max: 20–83 years)		
Marital status		
Married	173	79.7
Single	44	20.3
Education level		
Illiterate	20	9.2
Basic education	81	37.3
High school	82	37.8
University	34	15.7
Type of family		
Nuclear family	175	80.6
Extended family	42	19.4
Living with the patients		
Single	18	8.3
Family	189	87.1
Friends	5	2.3
Relatives	5	2.3
Working status		
Working	50	23.0
Nonworking	109	50.2
Emeritus	58	26.8
Place of residence		
City	101	46.5
District	63	29.0
Town/village	53	24.5
Perceived economic status		
Low incomes	161	74.2
Average incomes	49	22.6
High incomes	7	3.2
Status of being informed about the disease		
Yes	170	78.3
No	47	21.7
Supporters during the treatment process *		
Family	217	100.0
Friends	45	16.8
Nurse	64	23.9
Doctor	178	66.4
Volunteers	18	6.7
Other	7	2.6
Patients’ cancer type		
Endometrial carcinoma	108	49.8
Ovarian carcinoma	75	36.4
Cervical carcinoma	27	12.4
Vulvar carsinoma	4	1.8
Vaginal carsinoma	3	1.4
Patients’ time since diagnosis, year		
Less than 1	81	37.3
1–2	91	41.9
3	27	12.4
4	9	4.1
5 years and above	9	4.1
Patients’ stage of disease		
I	53	24.4
II	45	20.7
III	71	32.7
IV	17	7.8
No known stage	31	14.3
Medical treatment received		
Surgical	112	51.6
Chemotherapy	29	13.4
Radiotherapy	47	21.7
Hormone	14	6.5
Other	15	7.0
PSQI group		
Good sleep quality	66	32.6
Poor sleep quality	151	67.4

*More than one option is marked

## Distribution of the mean scores obtained by the participants from the scales

The distribution of the mean scores obtained by the participants from the scales is presented in Table 2. The patients’ total sleep quality score average was 6.91 ± 2.60. The total perceived social support score average was 59.85 ± 17.04. The social support scores averaged from family, friends, and special people were 23.37 ± 4.38, 17.56 ± 7.49, and 18.90 ± 7.62, respectively. The brief fatigue inventory total score average was determined as M (IQR) 63.00 (16.50) (Table 2).

**Table 2:** Distribution of the mean scores that participants received from the scales

Variables	*X* ± SD	*M* (IQR)
Subjective sleep quality	1.51 ± 0.87	
Sleep latency	1.71 ± 0.76	
Sleep time score		0.00 (0.00)
Sleep efficiency	1.74 ± 0.53	
Sleep disturbance	1.56 ± 0.65	
Sleep medication use	1.68 ± 0.45	
Daytime dysfunction	1.57 ± 0.77	
PSQI total	6.91 ± 2.60	
Perceived social support family	23.37 ± 4.38	
Perceived social support friends	17.56 ± 7.49	
Perceived social support special person	18.90 ± 7.62	
Perceived social support total	59.85 ± 17.04	
Brief fatigue ınventory total		63.00 (16.50)

### Social support and influencing factors

The comparison of participants’ social support scores based on their descriptive characteristics and medical data is presented in Table 3. The friends, special people, and total scale scores of participants living in the district centre were found to be statistically significantly lower (*p* < 0.05). The family subdimension scores of participants whose clinical stage of the disease was unknown were found to be statistically significantly higher than those of other subdimensions (*p* < 0.05). No statistically significant differences were found in social support scores based on the type of medical treatment received, other descriptive characteristics, or medical data (*p* > 0.05) (Table 3).

**Table 3:** The effect of various characteristics of the participants on the MPSSS^x^ and subscale scores

Characteristics	Family	Friends	Special person	Total
	*X* ± SD^y^ Test/p*	*X* ± SD^y^ Test/p*	X ± SD^y^ Test/p*	X ± SD^y^ Test/p*
Place of residence				
City a	23.51 ± 4.34	18.94 ± 7.58	20.11 ± 7.44	62.37 ± 17.57
District b	23.08 ± 4.39	14.77 ± 7.24	16.12 ± 7.75	54.06 ± 16.44
Town/village c	23.84 ± 4.07	18.16 ± 6.80	19.79 ± 7.05	61.81 ± 15.13
	*F* = 0.456	*F* = 6.565	*F* = 6.102	*F* = 5.314
	*p* = 0.654	*p* = 0.002	*p* = 0.003	*p* = 0.006
		*b* > ***a***,***c***	*b* > ***a***,***c***	*b* > ***a***,***c***
Patients stage of disease				
I a	23.21 ± 4.19	17.62 ± 7.64	18.33 ± 7.68	59.26 ± 16.93
II b	22.26 ± 4.35	17.11 ± 6.87	18.84 ± 6.70	58.22 ± 16.59
III c	23.04 ± 4.79	16.50 ± 6.75	18.82 ± 7.71	57.67 ± 16.44
IV d	24.29 ± 4.22	17.11 ± 5.09	11.07 ± 5.70	68.17 ± 18.10
No known stage e	25.54 ± 3.05	21.76 ± 7.76	22.11 ± 7.84	63.45 ± 18.14
	*F* = 3.045	*F* = 1.799	*F* = 1.125	*F* = 1.800
	*p* = 0.018	*p* = 0.130	*p* = 0.346	*p* = 0.130
	***e*** > ***a***,***b***,c,***d***			
Education status				
Primary	20.26 ± 4.31	18.66 ± 7.30	20.40 ± 7.19	62.70 ± 16.52
High school	22.71 ± 4.87	16.62 ± 7.35	17.39 ± 7.58	56.79 ± 16.99
University	24.61 ± 3.59	17.70 ± 7.80	19.35 ± 7.77	61.67 ± 17.09
Other	22.95 ± 3.36	16.50 ± 8.10	18.00 ± 8.41	57.45 ± 17.73
	*F* = 1.692	*F* = 1.159	*F* = 2.303	*F* = 1.930
	*p* = 0.170	*p* = 0.376	*p* = 0.078	*p* = 0.126
Marital status				
Married	23.14 ± 4.48	17.60 ± 7.52	18.68 ± 7.70	59.46 ± 17.25
Single	24.29 ± 3.88	17.31 ± 7.39	19.63 ± 7.30	61.25 ± 16.31
	*t* = − 1.559	*t* = 0.223	*t* = − 0.736	*t* = − 0.621
	*p* = 0.121	*p* = 0.823	*p* = 0.462	*p* = 0.535
Family type				
Nuclear	23.24 ± 4.39	17.81 ± 7.38	19.10 ± 7.42	60.18 ± 16.67
Extended	23.92 ± 4.35	16.42 ± 7.87	17.95 ± 8.40	58.30 ± 16.39
	*t* = − 0.904	*t* = 1.075	*t* = 0.878	*t* = 0.642
	*p* = 0.367	*p* = 0.283	*p* = 0.381	*p* = 0.282
Medical treatment received				
Surgical	23.27 ± 4.42	18.24 ± 7.43	19.47 ± 7.31	61.50 ± 16.69
Chemotherapy	23.68 ± 3.90	17.51 ± 7.22	19.06 ± 7.12	60.27 ± 16.61
Radiotherapy	22.82 ± 4.26	15.14 ± 7.76	16.91 ± 8.47	54.89 ± 16.53
Hormone	22.38 ± 8.94	19.21 ± 7.98	19.14 ± 8.65	62.28 ± 17.92
Other	22.38 ± 8.94	18.30 ± 6.31	20.07 ± 7.06	59.61 ± 18.35
	*F* = 1.344	*F* = 1.298	*F* = 0.866	*F* = 1.166
	*p* = 0.239	*p* = 0.259	*p* = 0.521	*p* = 0.326

*p < 0.05

^x^*MPSSS* multidimensional perceived social support scale

^y^*SD* standard deviation

### Fatigue and influencing factors

The comparison of the median fatigue scores of the participants according to their medical data is provided in Table 4. The fatigue scores of participants in the second and third stages of the disease were statistically significantly higher than those in other stages and those with an unknown stage. The fatigue scores of participants who received surgical treatment were statistically significantly lower than those who underwent other medical treatment methods (*p* < 0.05). No statistically significant differences were found in fatigue scores based on other medical data and descriptive characteristics (*p* > 0.05) (Table 4).

**Table 4:** The effect of clinical characteristics of the participants on the brief fatigue inventory score

Characteristics	Brief fatigue ınventory
	***M*** (IQR) Test/p*
Patients stage of disease	
I ^a^	50.00 (10.00)
II ^b^	66.00 (9.00)
III ^c^	65.00 (14.00)
IV ^d^	62.00 (41.00)
No known stage ^e^	56.00 (15.00)
	KW = 63.438
	*p* = 0.000
	*b*,*c* > *a*,*d*,*e*
Medical treatment received	
Surgical	56.50 (16.25)
Chemotherapy	64.00 (16.50)
Radiotherapy	63.00 (15.00)
Hormone	64.00 (13.25)
Other	66.00 (17.50)
	KW = 10.553
	*p* = 0.032
	*a* > *b*,c,d,*e*
Education status	
Primary	62.00 (21.00)
High school	63.00 (14.25)
University	63.50 (16.25)
Other	65.00 (15.75)
	KW = 0.642
	*p* = 0.887
Marital status	
Married	63.00 (16.00)
Single	59.50 (17.00)
	*U* = 3504.50
	*p* = 0.417
Family type	
Nuclear	62.00 (16.00)
Extended	64.00 (17.75)
	*U* = 3366.00
	*p* = 0.397

*p < 0.05

### Quality sleep and influencing factors

The comparison of the mean scores of PSQI according to the descriptive characteristics and medical data of the participants is provided in Table 5. A statistically significant difference was found in sleep duration among participants who lived with their relatives, those diagnosed with cervical or vaginal cancer, and those whose disease was at stage 4 (*p* < 0.05). The subjective sleep quality scores of participants with stage 4 disease were statistically significantly lower than those in other stages, while their daytime dysfunction scores were higher than those in other stages. Sleep disturbance and daytime dysfunction scores of those living in the district centre were statistically significantly higher (*p* < 0.05) (Table 5).

**Table 5:** The effect of various characteristics of the participants on the PSQI^x^ scores

Characteristics	Subjective sleep quality	Sleep latency	Sleep time		Sleep efficiency	Sleep disturbance		Sleep medication use		Daytime dysfunction	Total
	*X* ± SD^y^Test/*p*	*X*± SD^y^Test/*p*	*M*(IQR)^z^Test/*p*		*X*± SD^y^Test/*p*	*X*± SD^y^Test/*p*		*X*± SD^y^Test/*p*		*X*± SD^y^Test/*p*	*X*± SD^y^Test/*p*
Living with the patients											
Single ^a^	1.72 ± 0.95	2.00 ± 0.59	0.50/2.00		0.00 ± 0.00	1.66 ± 0.68		1.71 ± 0.48		1.72 ± 1.01	8.33 ± 3.32
Family ^b^	1.47 ± 0.88	1.70 ± 0.79	0.00/0.00		2.72 ± 0.46	1.55 ± 0.65		1.55 ± 0.92		1.56 ± 0.75	6.77 ± 2.54
Friends ^c^	1.80 ± 0.44	1.60 ± 0.54	0.00/0.00		2.57 ± 0.64	1.60 ± 0.54		1.71 ± 0.75		1.40 ± 0.54	7.00 ± 2.12
Relatives ^d^	2.00 ± 0.00 *F* = 1.146 *p* = 0.332	1.40 ± 0.54 *F* = 1.154 *p* = 0.328	0.00/0.00 KW = 11.323 *p* = 0.010 *d* > *a*,b,*c*		2.42 ± 0.70 *F* = 0.148 *p* = 0.931	1.80 ± 0.44 *F* = 0.398 *p* = 0.755		1.57 ± 0.70 *F* = 1.295 *p* = 0.277		2.00 ± 0.00 *F* = 0.824 *p* = 0.482	7.40 ± 0.54 *F* = 2.056 *p* = 0.107
Place of residence											
City ^a^	1.41 ± 0.93	1.75 ± 0.75	0.00/0.00		2.51 ± 0.69	1.49 ± 0.62		1.77 ± 0.71		1.50 ± 0.75	6.66 ± 2.65
District ^b^ Town/village ^c^	1.71 ± 0.79 1.47 ± 0.84 *F* = 2.365 *p* = 0.096	1.80 ± 0.82 1.54 ± 0.72 *F* = 1.868 *p* = 0.157	0.00/1.00 0.00/0.00 KW = 2.009 *p* = 0.366		2.60 ± 0.60 2.61 ± 0.50 *F* = 0.115 *p* = 0.892	1.77 ± 0.60 1.45 ± 0.69 *F* = 4.920 *p* = 0.008 *b* > *a*,*c*		1.57 ± 0.79 1.53 ± 0.77 F = 0.018 *p* = 0.982		1.82 ± 0.77 1.43 ± 0.74 *F* = 4.765 *p* = 0.009 *b* > *a*,*c*	7.73 ± 2.43 6.45 ± 2.53 *F* = 4.553 *p* = 0.012 *b* > *a*,*c*
Patients cancer type											
Endometrial carcinoma^a^	1.42 ± 0.94	1.70 ± 0.82		0.00/0.75	2.17 ± 0.72	1.52 ± 0.64	1.23 ± 0.66		1.59 ± 0.79		6.79 ± 2.71
Ovarian carcinoma ^b^ Cervical carcinoma ^c^ Vulvar carsinoma ^d^ Vaginal carsinoma ^e^	1.58 ± 0.79 1.59 ± 0.79 2.00 ± 1.15 1.66 ± 0.57 *F* = 0.781 p = 0.538	1.69 ± 0.73 1.85 ± 0.71 2.00 ± 0.00 1.33 ± 0.57 *F* = 0.550 *p* = 0.699		0.00/0.00 0,00/0,00 2.00/1.50 0.00/0.00 KW = 13.871 *p* = 0.008 *c*,*e* > *a*,*b*,*d*	2.32 ± 0.74 2.80 ± 0.45 2.65 ± 0.57 2.63 ± 0.58 *F* = 4.797 *p* = 0.001 *d* > *a*,*b*,*c*,*e*	1.61 ± 0.67 1.62 ± 0.56 1.50 ± 1.00 1.33 ± 0.57 *F* = 0.852 *p* = 0.494	1.54 ± 0.80 1.78 ± 0.68 1.80 ± 0.75 1.78 ± 0.68 *F* = 0.359 *p* = 0.837		1.52 ± 0.77 1.74 ± 0.59 1.25 ± 1.25 1.66 ± 0.57 *F* = 0.600 *p* = 0.663		7.02 ± 2.60 7.00 ± 2.09 8.50 ± 3.69 6.00 ± 1.00 *F* = 0.556 *p* = 0.695
Patients’ stage of disease											
I ^a^	1.37 ± 0.85	1.62 ± 0.92		0.00/0.00	2.69 ± 0.56	1.49 ± 0.63	1.78 ± 0.71		1.52 ± 0.78		6.70 ± 2.53
II ^b^	1.77 ± 0.67	1.62 ± 0.74		0.00/0.00	2.33 ± 0.72	1.62 ± 0.61	1.60 ± 0.98		1.75 ± 0.68		7.62 ± 2.38
III ^c^	1.56 ± 0.82	1.81 ± 0.59		0.00/0.00	2.38 ± 0.70	1.60 ± 0.54	1.46 ± 0.71		1.59 ± 0.77		6.97 ± 2.61
IV ^d^	1.00 ± 1.11	1.82 ± 0.80		0.00/2.00	1.49 ± 0.76	1.52 ± 0.94	1.58 ± 0.82		1.78 ± 0.69		7.21 ± 3.68
No known stage ^e^	1.54 ± 1.02	1.74 ± 0.85		0.00/1.00	1.55 ± 0.72	1.54 ± 0.76	1.56 ± 0.84		1.38 ± 0.86		6.76 ± 2.38
	*F* = 2.971	*F* = 0.755		KW = 12.787	*F* = 1.166	*F* = 0.343	*F* = 1.468		*F* = 3.613		*F* = 0.927
	*p* = 0.020	*p* = 0.556		*p* = 0.012	*p* = 0.327	*p* = 0.848	*p* = 0.213		*p* = 0.007		*p* = 0.449
	*d* > *a,b*,*c*,*e*			*d* > *a*,*b*,*c*,*e*					*d* > *a*,*b*,*c*,*e*		

^x^*PSQI* Pittsburg Sleep Quality Index

^*y*^*SD* standard deviation

^*Z*^*IQR* interquartile range

#### Correlation analysis of sleep quality, social support, and fatigue

In Table 6, the relationship between the scales and sub-dimension mean scores was evaluated by correlation coefficient (*r*), and *r* < 0.3 was expressed as a weak relationship, 0.3 < *r* < 0.7 as a moderate relationship, and 0.7 < *r* < 1 as a strong relationship. There was a weak negative correlation between the subjective sleep quality sub-dimension and social support scale sub-dimensions and total score and a weak positive correlation with the fatigue scale. A negative weak relationship was found between the sleep latency sub-dimension and social support friends, special people, and the total score of the scale. There was a weak positive correlation between sleep duration and social support friends, special people, and scale total score. There was a moderate negative correlation between the sleep disturbance sub-dimension and social support friends, special people and scale total score, and a weak positive correlation with the fatigue scale. There was a weak negative correlation between the sub-dimension of daytime dysfunction and the total score of PSQI and the sub-dimensions and total score of the social support scale and a weak positive correlation with the fatigue scale (*p* < 0.05) (Table 6).

**Table 6:** Correlation analysis of sleep quality, social support, and fatigue

Variables		1	2	3	4	5	6	7	8	9	10	11	12	13
(1) Subjective sleep quality	*r*	*-*												
	*p*	*-*												
(2) Sleep latency	*r*	0.353	-											
	*p*	0.000	-											
(3) Sleep time	*r*	− 0.037	0.173	-										
	*p*	0.588	0.011	-										
(4) Sleep efficiency	*r*	− 0.070	0.043	0.368	-									
	*p*	0.306	0.525	0.000	-									
(5) Sleep disturbance	*r*	0.516	0.413	0.044	− 0.043	-								
	*p*	0.000	0.000	0.521	0.532	-								
(6) Sleep medication use	*r*	− 0.002	− 0.094	0.345	− 0.046	0.156	-							
	*p*	0.972	0.425	0.000	0.505	0.022	-							
(7) Daytime dysfunction	*r*	0.670	0.292	− 0.100	− 0.140	0.549	− 0.003	-						
	*p*	0.000	0.000	0.142	0.039	0.000	0.964	-						
(8) PSQI^a^ total	*r*	0.755	0.629	0.362	0.064	0.751	0.311	0.713	-					
	*p*	0.000	0.000	0.000	0.346	0.000	0.000	0.000	-					
(9) Perceived social support family	*r*	− 0.161	− 0.041	0.020	0.062	− 0.105	−0.045	− 0.250	− 0.168	-				
	*p*	0.018	0.551	0.766	0.364	0.124	0.513	0.000	0.013	-				
(10) Perceived social support friends	*r*	− 0.338	− 0.150	0.171	0.050	− 0.469	0.102	− 0.375	− 0.333	0.396	-			
	*p*	0.000	0.028	0.012	0.468	0.000	0.133	0.000	0.000	0.000	-			
(11) Perceived social support special person	*r*	− 0.365	− 0.196	0.177	− 0.085	− 0.426	0.112	− 0.407	− 0.322	0.421	0.900	-		
	*p*	0.000	0.004	0.009	0.413	0.000	0.101	0.000	0.000	0.000	0.000	-		
(12) Perceived social support total	*r*	− 0.353	− 0.162	0.159	0.076	− 0.423	− 0.083	− 0.411	− 0.333	0.620	0.944	0.951	-	
	*p*	0.000	0.017	0.019	0.267	0.000	0.223	0.000	0.000	0.000	0.000	0.000	-	
(13) Brief fatigue ınventory total	*r*	0.269	0.063	0.050	− 0.029	0.222	0.080	0.304	0.283	− 0.117	− 0.097	− 0.111	− 0.124	-
	*p*	0.000	0.153	0.419	0.673	0.001	0.241	0.000	0.000	0.086	0.156	0.102	0.68	-

^a^ PSQI, Pittsburg Sleep Quality Index

## Discussion

Gynecological cancer patients’ relationships with their family, friends, and social environment may deteriorate due to prolonged treatments [8, 24]. Social support is very important for patients to overcome these problems. In our study, the perceived social support levels of women were found to be high and it was determined that they received more social support from the family. In the study of Uslu Şahan et al. [25], the social support perceived by gynecological oncology patients was found to be quite high. In other studies conducted in gynecological oncology patients, family support was found to be high [26–30]. In Turkish society, it is an indispensable part of the culture that family members support each other and stand by each other. In addition, it is common in Turkey for non-family members such as neighbors, relatives, and friends to help each other in complex life events such as illness. Therefore, Turkish family structure, cultural characteristics, and demographic characteristics of the participants may also influence the study.

In the current study, fatigue scores of patients with gynecological cancer were found to be above the middle level. In the literature, it is reported that patients experience high rates of cancer-related fatigue. Around 55–86.7% of women with gynecological cancer undergoing chemotherapy or chemoradiotherapy report experiencing cancer-related fatigue [28–30]. In a study conducted with individuals with different cancer types and stages in the literature, it was reported that the mean fatigue severity score of cancer patients was low [31]. In Özkan and Akın’s [32] study, it was determined that cancer patients undergoing chemotherapy experienced moderate fatigue. Mota et al. [33] reported that half of the patients experienced fatigue and the mean fatigue level score of patients with fatigue was high. In addition, it has been reported that cancer-related fatigue is the most frequently expected side effect of cancer treatment and 95% of patients who are planning to receive chemotherapy or radiotherapy are expected to experience fatigue during their treatment [14]. The results of our study were found to be similar to the studies. In gynecological cancers, the stage of the disease and the treatment received may cause increased fatigue. Nurses working in this field should evaluate the stage of the disease and the complications of the treatment received and plan nursing care.

In this study, poor sleep quality was found in most of the participants. In the study by Chen et al. [15], 42.5% of the participants reported poor sleep quality and sleep disturbance. Edmed et al. [34] reported that over half of the participants (52.6% to 56.7%) had poor sleep quality, efficiency, and duration. The stage of the disease, the treatments received, and the side effects of these treatments may have affected the sleep quality of the patients.

In our study, social support friends, special people, and total scale scores of those living in the district center were found to be statistically significantly lower. In the study of Çalışkan et al. [35], it was found that the mean total scores of the social support scale of patients living in the city were higher than those living in the village. It may be considered that the mutual support among individuals living in smaller settlements influences the nature of their social interactions.

In this study, the family subdimension scores of participants whose clinical stage of the disease was unknown were found to be higher than those of the other subdimensions. The stage of gynecological cancer affects the psychological response of individuals to the disease. In a study conducted by Liu et al. [36] with women with ovarian cancer, it was found that women with stage III and IV cancer showed higher levels of posttraumatic stress disorder symptoms compared to those with stages I and II, and there was a negative relationship between social support and posttraumatic stress disorder symptoms. In another study conducted with women diagnosed with ovarian cancer, it was reported that those with advanced-stage cancer had higher levels of social support compared to those with early-stage cancer [37]. This may be influenced by the Turkish family structure, cultural characteristics, and the demographic profile of the participants.

In our study, no statistical significance was found between the medical treatment received and social support scores. In a study conducted by Scheepers et al. [38] with women with ovarian cancer, it was reported that the level of perceived social support among women did not vary based on the type of treatment. Since the sample group consisted of women undergoing treatment in the hospital, being away from their own living environments and social support systems may have contributed to this outcome.

In the current study, the fatigue scores of participants in the second and third stages were statistically significantly higher than those in other stages and those with an unknown stage. In the literature, there are studies supporting that the severity of fatigue perceived by individuals with high cancer stage is higher than other stages [11, 31, 39, 40]. In contrast to this study, in a study conducted by Lavdaniti et al. [41], it was concluded that the stage of the disease did not affect the severity of fatigue. It is believed that the intensification of treatments as the disease progresses contributes to an increase in fatigue levels. Improving social support levels of patients helps them continue their cope with efforts during long and intensive treatment periods.

In our study, the fatigue scores of participants who received surgical treatment were statistically significantly lower than those who received other medical treatment modalities. Banipal et al. [11] reported in their study analyzing the prevalence of cancer-related fatigue in cancer patients and its correlation with treatment modalities that they found statistical correlations between fatigue and chemotherapy agents such as vinblastine, dacarbazine, and cyclophosphamide. They also stated that cancer-related fatigue is a symptom experienced by the majority of cancer patients, regardless of their diagnosis or type of treatment [39]. It is known that cancer patients receiving chemotherapy have increased fatigue levels during the treatment process [39, 41]. The results of our study were similar to those of other studies, but no drug-based evaluation was made because of the heterogeneous data due to the use of different cancer types and different chemotherapeutic agents.

In this study, it was determined that the sleep scores of the patients according to the medical treatment they received were not statistically significant. The majority of sleep problems occur after surgical intervention. Surgical stress includes sleep fragmentation and reduced sleep duration due to pain and postoperative medications [17, 42]. Depressive symptoms may lead to decreased sleep over time in ovarian cancer patients [43]. In the study by Beesley et al. [12], it was stated that while more than 50% of women regain their health status within 6 months, they continue to experience symptoms that affect their sleep and overall health for more than 18 months after treatment. Sleep problems are often reported after cancer-related surgery, including cases of ovarian cancer [44].

In this study, sleep duration was found to be lower in patients who lived with their relatives, had a medical diagnosis of cervical or vaginal cancer, and whose disease was at stage IV. Patients with gynecological cancer were more likely to suffer from poor sleep quality than patients with other types of cancer (gynecological cancer: 74.0%, head and neck cancer: 54.7%, breast cancer: 51.8%, prostate cancer: 50.7%, colorectal cancer: 47.9%, lung cancer: 47.3%). Several factors may be associated with the high rate of poor sleep quality in patients with cancer. Firstly, chemotherapeutic agents harm both neoplastic and normal cells, leading to common side effects such as nausea and vomiting, myelosuppression (anemia and leukopenia), renal and hepatic hypofunction, neurotoxicity, and cardiotoxicity [45, 46], all of which may impact sleep quality in cancer patients. Secondly, patients with cancer often face substantial psychosocial stress. Due to the tedious and challenging treatment process, negative emotions in patients with cancer are inevitable [13]. Thirdly, cancer can directly affect the regulation of the sleep circuit, resulting in disrupted homeostatic, hypothalamic, and circadian regulation of the sleep center in the brain [47]. After a cancer diagnosis, patients often expect attention and support from their families and may experience disappointment if they do not receive the support they anticipate. In addition, it has been determined that loneliness in cancer patients leads to issues such as weakened immune function, increased depression, fatigue, pain, and sleep disorders [48]. Adams et al. [49] found that loneliness was positively associated with fatigue and pain and negatively associated with sleep quality in people who were diagnosed with vaginal cancer and who had recently survived cancer. These factors may have negatively affected the sleep quality of patients in the fourth stage of the disease.

In the study of Sukyati et al. [50], it was concluded that social support from family, friends, and important people greatly influenced psychosocial effects such as decreasing depression and increasing cancer acceptance, having good coping mechanisms to cope with stress, increasing quality of life, and decreasing anxiety levels. In addition, this study stated that a lack of social support may lead to feelings of loneliness and loss of hope. Insomnia in patients diagnosed with ovarian cancer can affect 14–60% of patients [43]. In this study, sleep disturbance and daytime dysfunction scores of those living in the district center were statistically significantly higher. A meta-analysis revealed that a higher prevalence of poor sleep quality was reported in studies conducted in the Middle East and North Africa region and low-income countries and in studies on gynecological cancer [15]. Not residing in a provincial center may act as a stressor in managing cancer and its symptoms, potentially contributing to sleep disturbances in women diagnosed with cancer.

In the current study, it was found that subjective sleep quality decreased slightly, while fatigue levels increased slightly as social support increased. As social support increased, sleep latency and sleep duration also increased; sleep disturbances decreased moderately, and daytime dysfunctions decreased slightly. It is stated in the literature that cancer patients frequently experience sleep problems [51]. Adequate and quality sleep relaxes both the body and mind, activates the parasympathetic nervous system, and helps relax the muscles in the body. A good sleep habit helps individuals to regulate their biological rhythms. For this reason, adequate and quality sleep can enhance physical strength, strengthen the immune system, and help reduce the physical and cognitive fatigue experienced by the individual [51, 52]. In studies conducted in the literature, it is reported that sleep problems affect the fatigue level of individuals [51, 52]. In addition, there are studies in the literature indicating that sleep problems do not fully account for the fatigue experienced by individuals [15, 27]. It is believed that restricting daytime sleep, reducing the total time spent in bed, establishing a regular sleep schedule, and promoting sleep hygiene will reduce the level of fatigue experienced by individuals. In a study of patients with endometrial cancer, it was reported that there was a strong relationship between sleep quality and fatigue [42]. Haque et al. [53] found that fatigue was six times higher in patients with insomnia symptoms. In their study [39], Mardanian-Dehkordi and Kahangi stated that social support contributed to improvements in the physical dimension of fatigue, and as patients’ social support increased, their physical fatigue decreased. They also found that while the majority of the patients received emotional support, regression analysis revealed that emotional support was not effective in reducing fatigue [39].

## Limitations

The study was conducted only in one region of Turkey. The study was limited to women in the Gynaecological Oncology Clinic of a Training and Research Hospital, and the results provide information about only one group. Therefore, the study results cannot be generalized to the general population. Therefore, the study results cannot be generalized to the broader population.

## Conclusion

In this study, perceived social support and fatigue scores among patients with gynecological cancer were found to be above the moderate level, while their sleep quality was poor. In our study, patients with second- and third-stage gynecological cancer had higher fatigue scores, while those who received surgical treatment had lower fatigue scores. As perceived social support increased, sleep latency and sleep duration increased; sleep disturbances decreased moderately and daytime dysfunctions decreased poorly. In line with these results, nurses should assess patients for fatigue and sleep quality, while also taking social support systems into consideration during the evaluation. Nurses should aim to identify and strengthen the social support systems of patients, plan care using a multidisciplinary team approach, and ensure coordination among other team members throughout the process. Care interventions can be organized in a way that does not interfere with patients’ sleep and rest hours. Since effective management of cancer-related symptoms positively influences sleep quality and fatigue, oncology nurses may need to receive training in symptom management. It is recommended that randomized controlled studies examining the effect of nursing interventions on sleep quality, social support, and fatigue in patients with gynecological cancer be conducted, and that the literature on this subject be expanded.

## Data Availability

No datasets were generated or analysed during the current study.

## Code availability

Statistical analyses were conducted using the SPSS version 22.0 and SAS version 9.4 software packages.
